# Supplementary material for: Structural insights into the mechanism of GTP initiation of microtubule assembly
Source: Nat Commun. 2023 Sep 25;14:5980. doi: 10.1038/s41467-023-41615-w (PMC10519996; doi:10.1038/s41467-023-41615-w)
Supplement: Supplementary file 1 — Supplementary Information [file 41467_2023_41615_MOESM1_ESM.pdf]

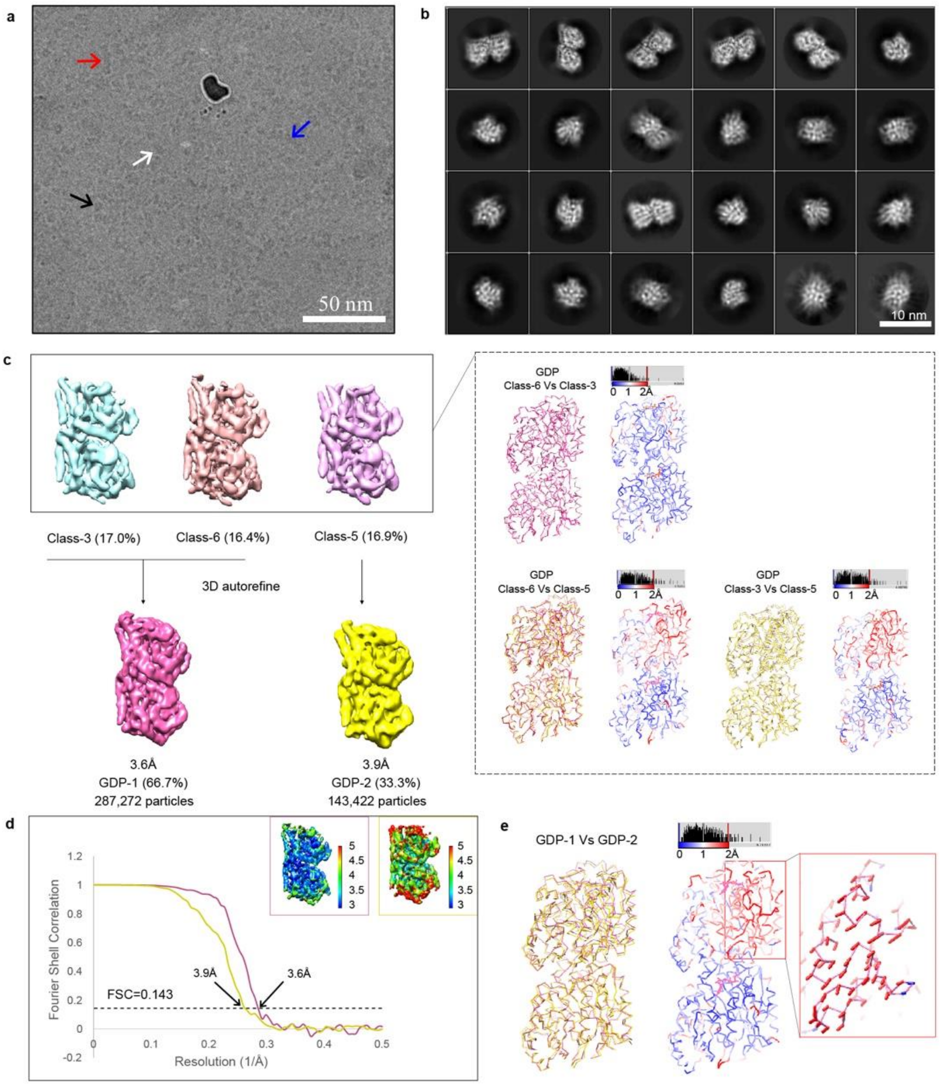

Supplementary Fig. 1 The workflow of Cryo-EM structure reconstruction of GDP-tubulin heterodimer

a A representative cryo-EM micrograph of GDP-tubulin sample. The side view and top view of tubulin dimer particles are marked with white and black arrows, respectively. The tubulin tetramer and hexamer particles are indicated by red and blue arrows, respectively.

b 2D classification results of GDP-tubulin heterodimer particles.

c Flowcharts for 3D classification and refinement. Based on the structural comparison and RMSD analysis of tubulin heterodimer model in the dashed box to the right, Class-3 and Class-6 particles are combined into one class (termed GDP-1 state). Class-5 is referred to as GDP-2 state.

d Corrected Fourier Shell Correlation (FSC) curves at 0.143 criterion of the final 3D reconstruction of GDP-1 (pink) and GDP-2 (yellow) state. The corresponding local resolution estimation maps are shown in the inset.

e Structural comparison (left) and RMSD analysis (right) of Cα traces between GDP-1

and GDP-2 state. The chain-trace displayed corresponds to the GDP-1 model. The inset shows the displacement of H2-S3 and H1-S2 loop. The vector greater than 1Å is colored in red and less than 1Å is colored in blue.

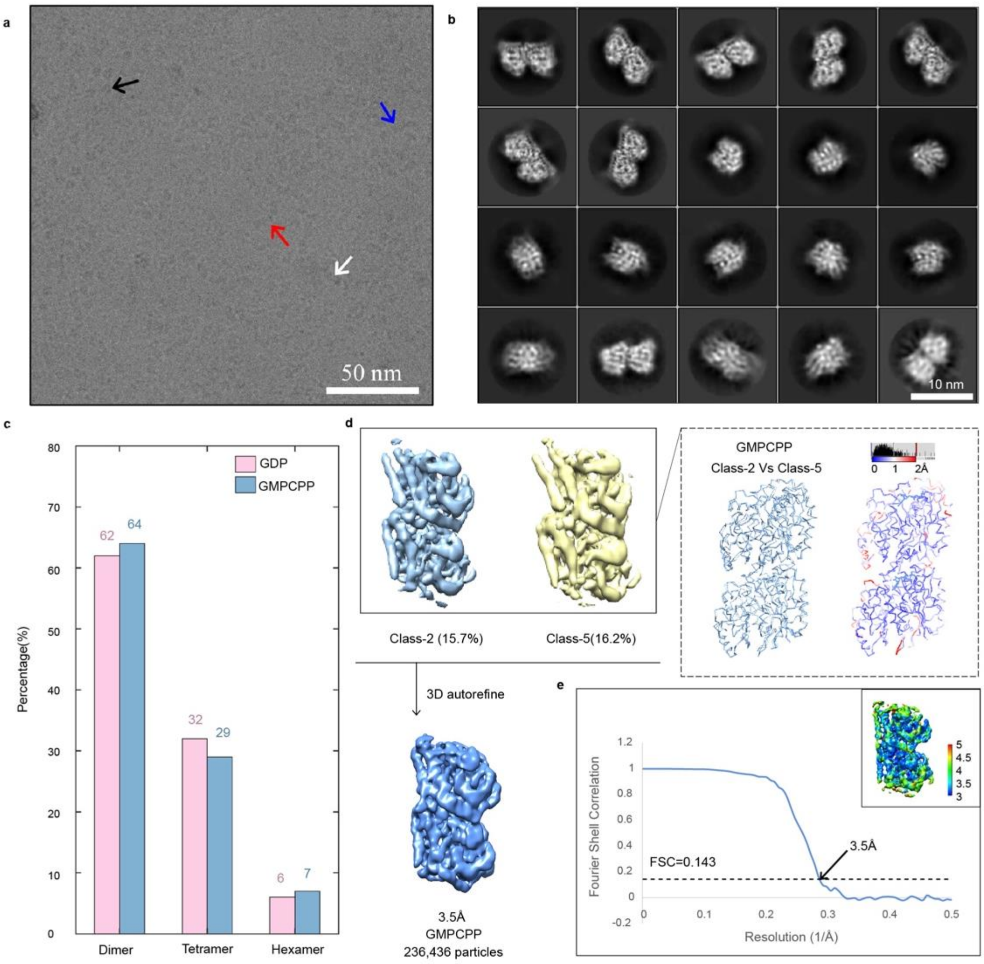

Supplementary Fig. 2 The workflow of Cryo-EM structure reconstruction of GMPCPP-tubulin heterodimer

a A representative cryo-EM micrograph of GMPCPP-tubulin sample. The side view and top view of tubulin dimer particles are marked with white and black arrows, respectively. The tubulin tetramer and hexamer particles are labeled with red and blue arrows, respectively.

b 2D classification results of GMPCPP-tubulin heterodimer particles.

c The percentage of tubulin dimers, tetramers, and hexamers in the total number of particles. The vertical axis displays the percentage value, while the horizontal axis displays the types of tubulin particles. The GDP state is colored in pink, while the GMPCPP state is colored in blue.

d Flowcharts of 3D classification and refinement. Combining Class-2 with Class-5 particles into one final class is based on the structural comparison and RMSD analysis of tubulin heterodimer model in the dashed box on the right.

e Corrected Fourier Shell Correlation (FSC) curve at 0.143 criterion of the final 3D reconstruction of GMPCPP-tubulin heterodimer. The corresponding local resolution

estimation map is shown in the inset.

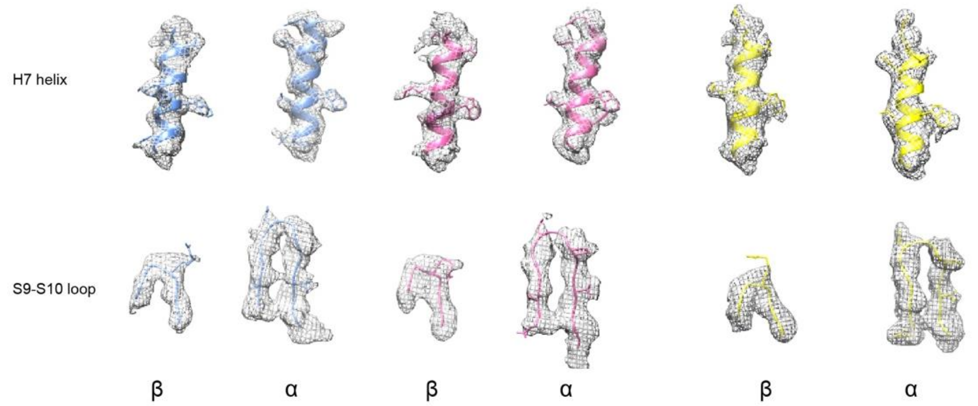

Supplementary Fig. 3 Cryo-EM density maps of representative segments and corresponding models

H7 helix is displayed on the top and S9-S10 loop is demonstrated at the bottom. The models in GMPCPP, GDP-1 and GDP-2 states are colored with cornflower blue, hot pink and yellow.

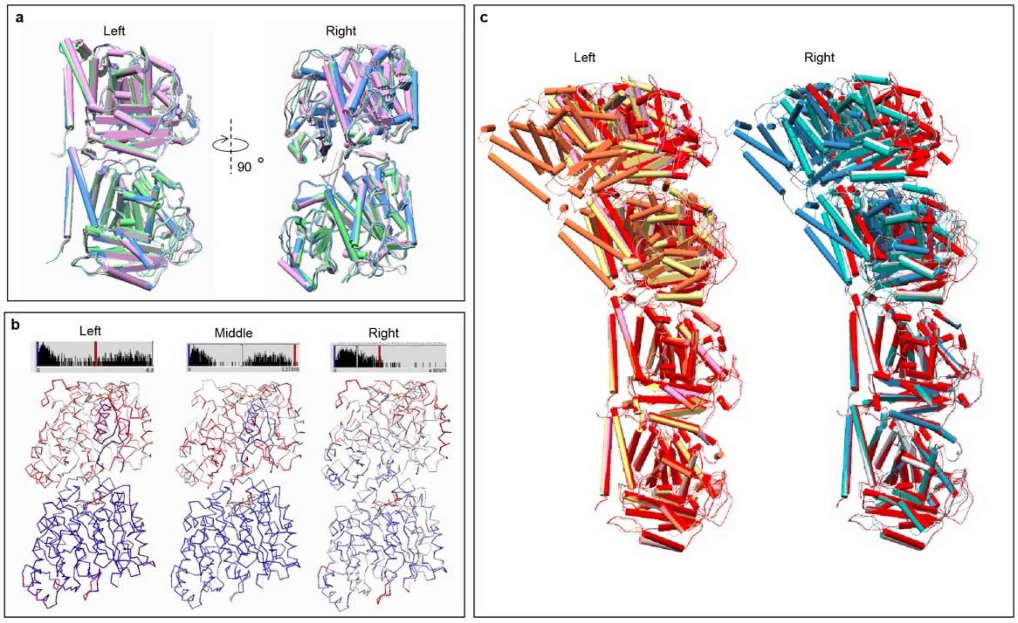

Supplementary Fig. 4 Structural comparison of tubulin heterodimer and tetramer solved by cryo-EM and X-ray crystallography

a Structural comparison between GTP-tubulin heterodimers determined by cryo-EM and X-ray crystallography, superimposing on the α-tubulin. The cryo-EM structure is colored with blue, the crystal structures are colored with plum (the lower one in PDBID: 6tiy), green (the upper one in PDBID: 6tiy) and light grey (PDBID: 4drx). Left: Side View. Right: Back view.

b Cα-RMSD analysis of tubulin heterodimers in (**a**). Left: Cα-RMSD analysis between

cryo-EM structure and crystal structure (the lower one in PDBID: 6tiy). Middle: Cα-RMSD analysis between cryo-EM structure and crystal structure (the upper one in PDBID: 6tiy). Right: Cα-RMSD analysis between cryo-EM structure and crystal structure (PDBID: 4drx).

c Overview of three major conformations of GDP- and GMPCPP-tubulin tetramers. All models are aligned together using the lower β-tubulin as reference. The crystal structure of tubulin tetramer (PDBID:6tiy) is colored in red. Left: GDP-tubulin tetramers are colored with coral, plum and khaki, respectively. Right: GMPCPP-tubulin tetramers are colored with steel blue, light sea green and light grey, respectively.

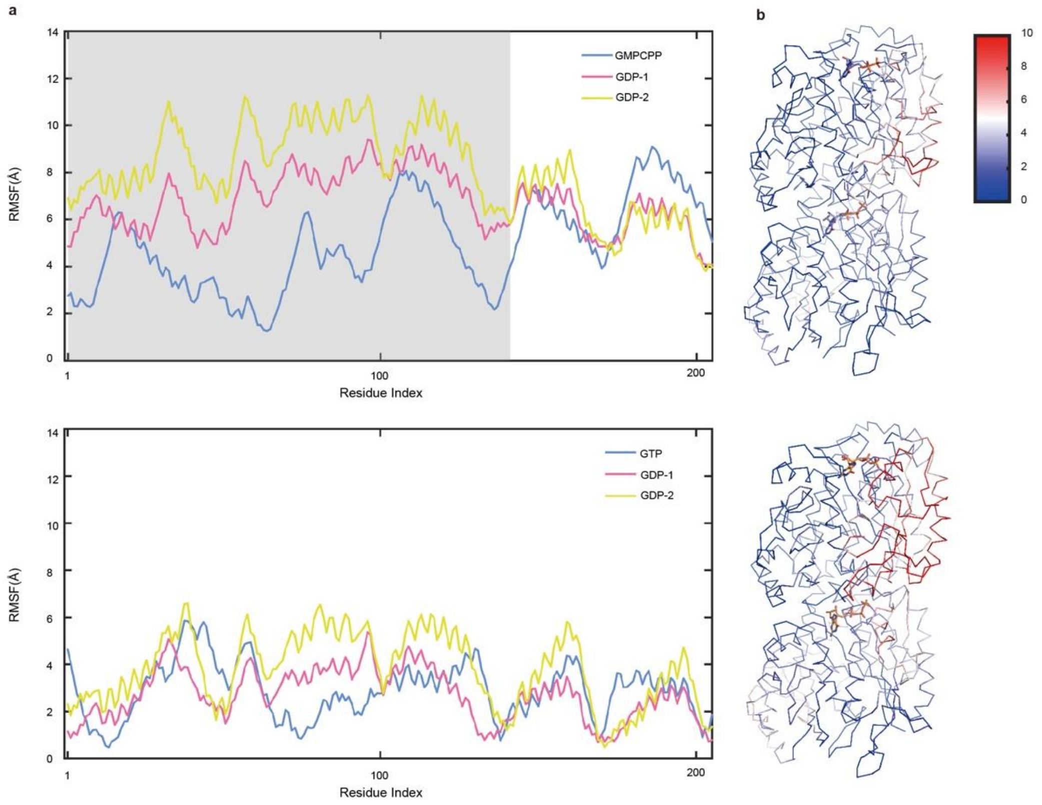

Supplementary Fig. 5 RMSF analysis of tubulin heterodimer models between GDP and GTP states

a The Cα-atoms-RMSF value distribution of the N-terminal (1-205 amino acids) of GDP-1 (pink), GDP-2 (yellow) and GTP (blue) tubulin heterodimer models. Top: β-tubulin. Shaded areas show the RMSF values of the N-terminal 1-120 amino acids near the γ-phosphate side. Bottom: α-tubulin.

b Top: Cα-atoms-RMSF analysis between GDP-1 and GTP tubulin heterodimer models. Bottom: Cα-atoms-RMSF analysis between GDP-2 and GTP tubulin heterodimer models. Atom structures are shown as stick diagrams, with color bars indicating RMSF values in Angstroms.

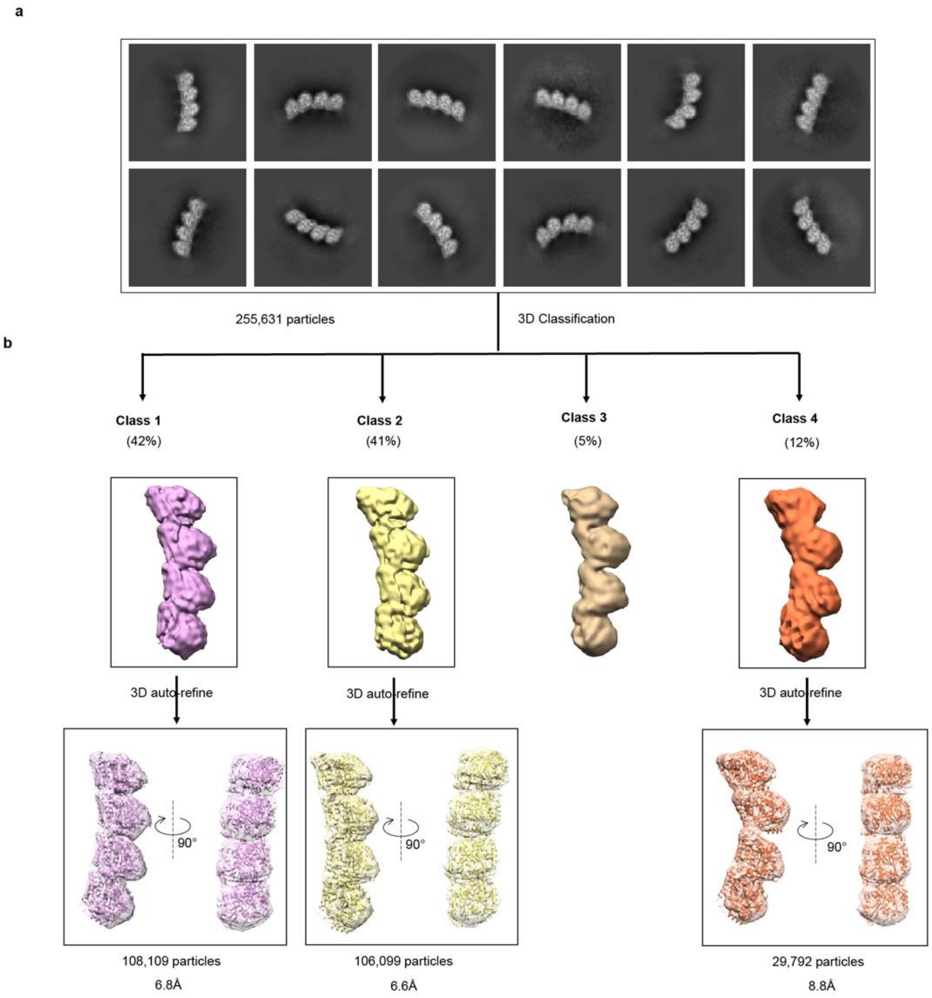

Supplementary Fig. 6 The workflow of Cryo-EM structure reconstruction of GDP-tubulin tetramer

a 2D classification results.

b 3D classification and refinement.

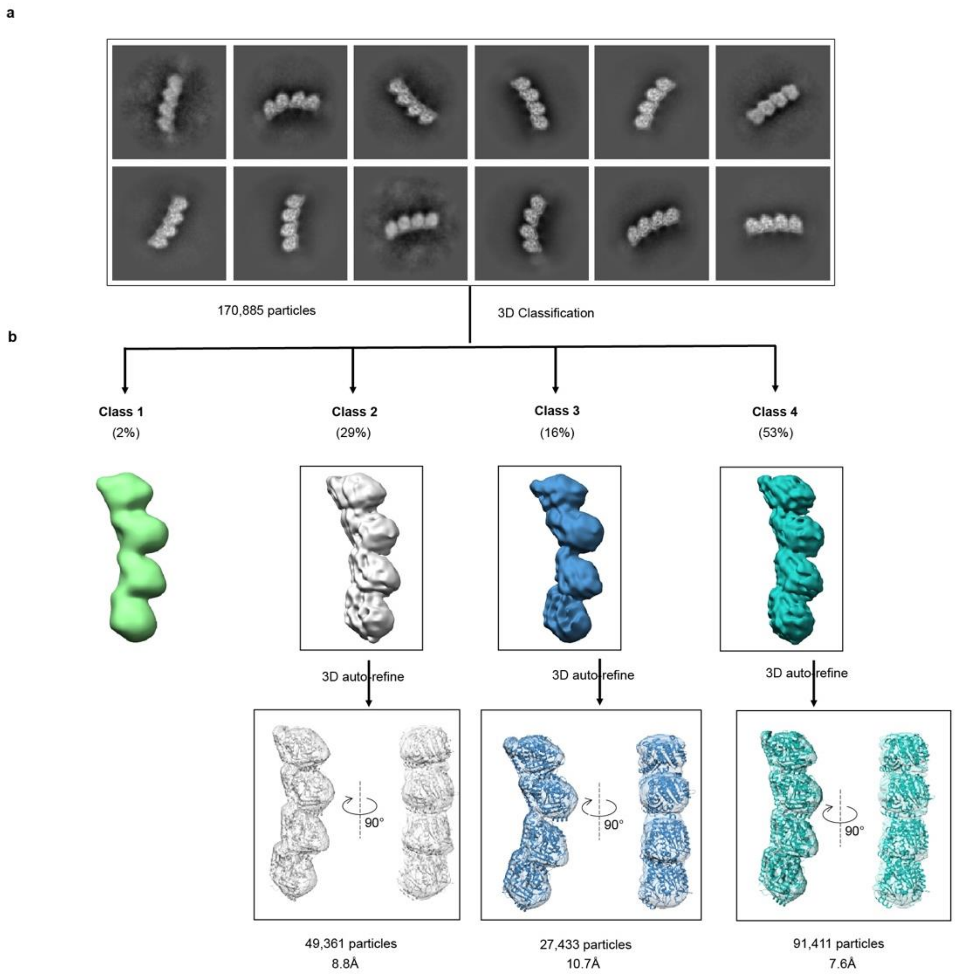

Supplementary Fig. 7 The workflow of Cryo-EM structure reconstruction of GMPCPP-tubulin tetramer

a 2D classification.

b 3D classification and refinement.

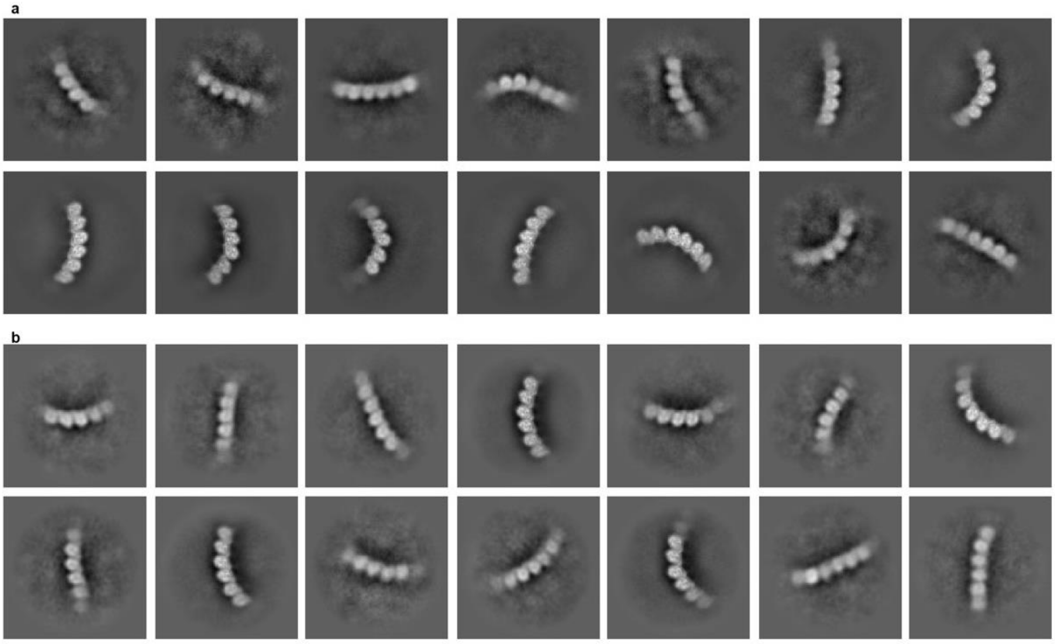

Supplementary Fig. 8 The 2D classification result of tubulin hexamer particles
a The 2D classification result of GDP state. **b** The 2D classification result of GMPCPP state.

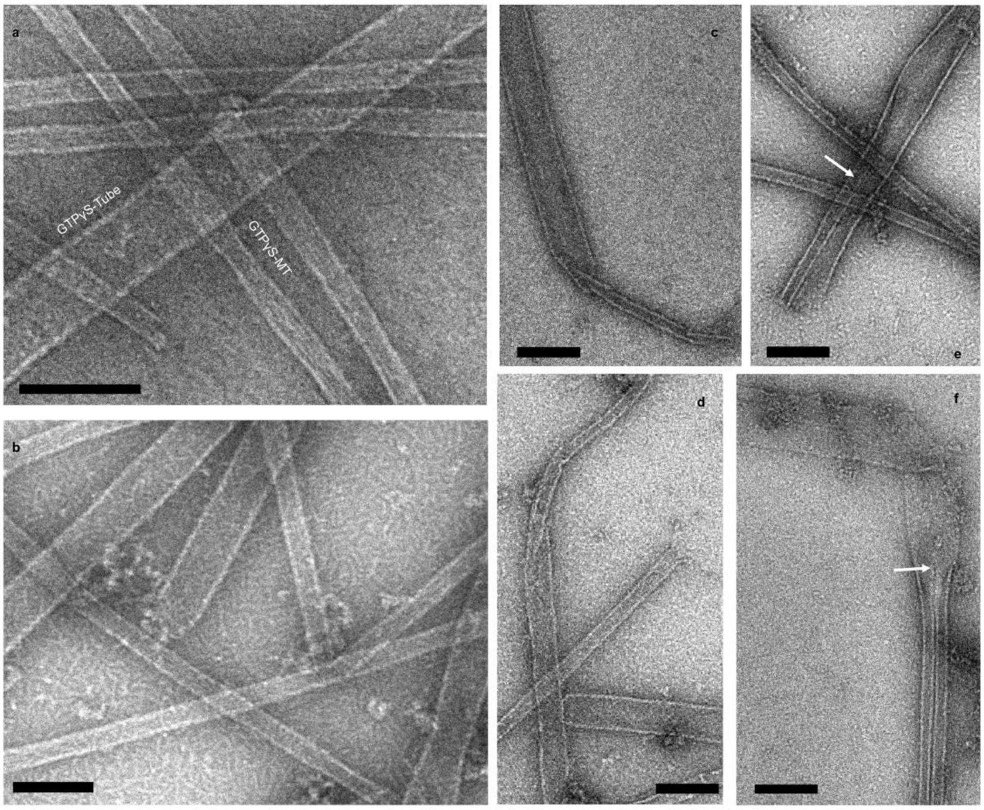

Supplementary Fig. 9 Negative-staining EM micrographs of coexistence and conversion of GTPγS-Tubes and MTs

a GTPγS-Tube and MTs at 2mM Mg^{2+}. The wider GTPγS-Tube and narrower GTPγS-MT are labeled respectively. Scale bar: 100 nm (same for **b-f**).

b GTPγS-Tube co-exist with MT at 5mM Mg^{2+}.

c-f GTPγS-Tube converts into MT directly in two different ways. **c-d**: One Tube converts into one MT (Conversion-1). **e-f** One Tube splits into two MTs (Conversion-2). The branch point is pointed out by the white arrow.

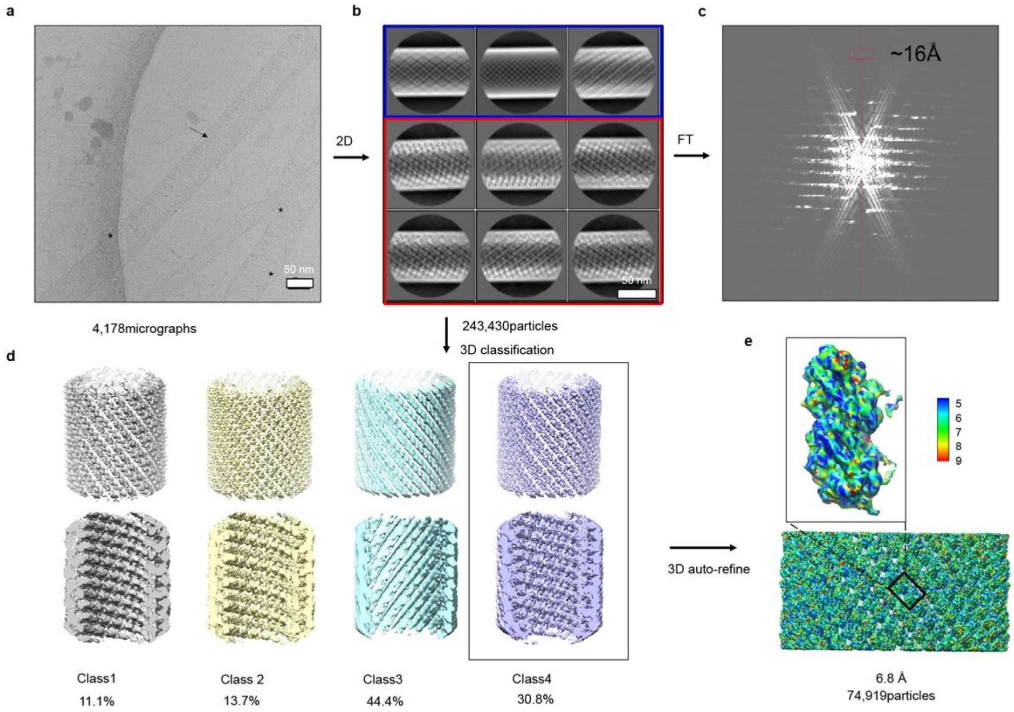

Supplementary Fig. 10 The workflow of Cryo-EM structure reconstruction of GTPγS-Tube decorated by KMD.

a A representative cryo-EM micrograph of GTPγS-Tube decorated by KMD. The arc-shaped stripes indicate KMD binding (marked by the black arrow). The broken areas of the GTPγS-Tube are marked with asterisks.

b 2D classification results. The red rectangle indicates good classes and the blue rectangle indicates bad classes.

c The power spectrum of one of the selected class labeled with red rectangle in **b**. A layer line at 16 Angstrom is marked.

d 3D classification results. An outside view is shown on top, and an inside view is shown on the bottom. Further refinement is selected for Class-4 because it has better structural details.

e 3D auto-refinement and local resolution map estimation.

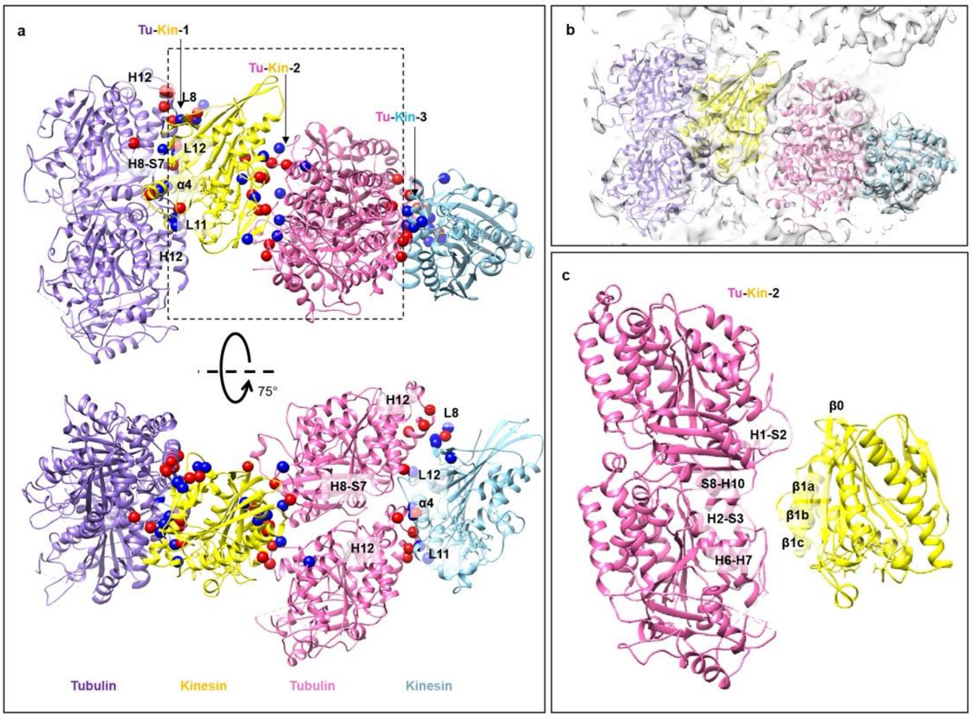

Supplementary Fig. 11 Tubulin-kinesin interaction interfaces in the GTPγS-Tube-KMD.

a Three tubulin-kinesin interaction interfaces in the GTPγS-Tube-KMD complex are labeled. Secondary structures involved in interface-1 and 3 are marked. Positively and negatively charged residues around the interfaces are represented as small blue and red spheres.

b The cryo-EM density map docked well with the tubulin and KMD models.

c Zoom-in view of Tu-Kin-2 interface, secondary structures engaged in this interaction are marked.

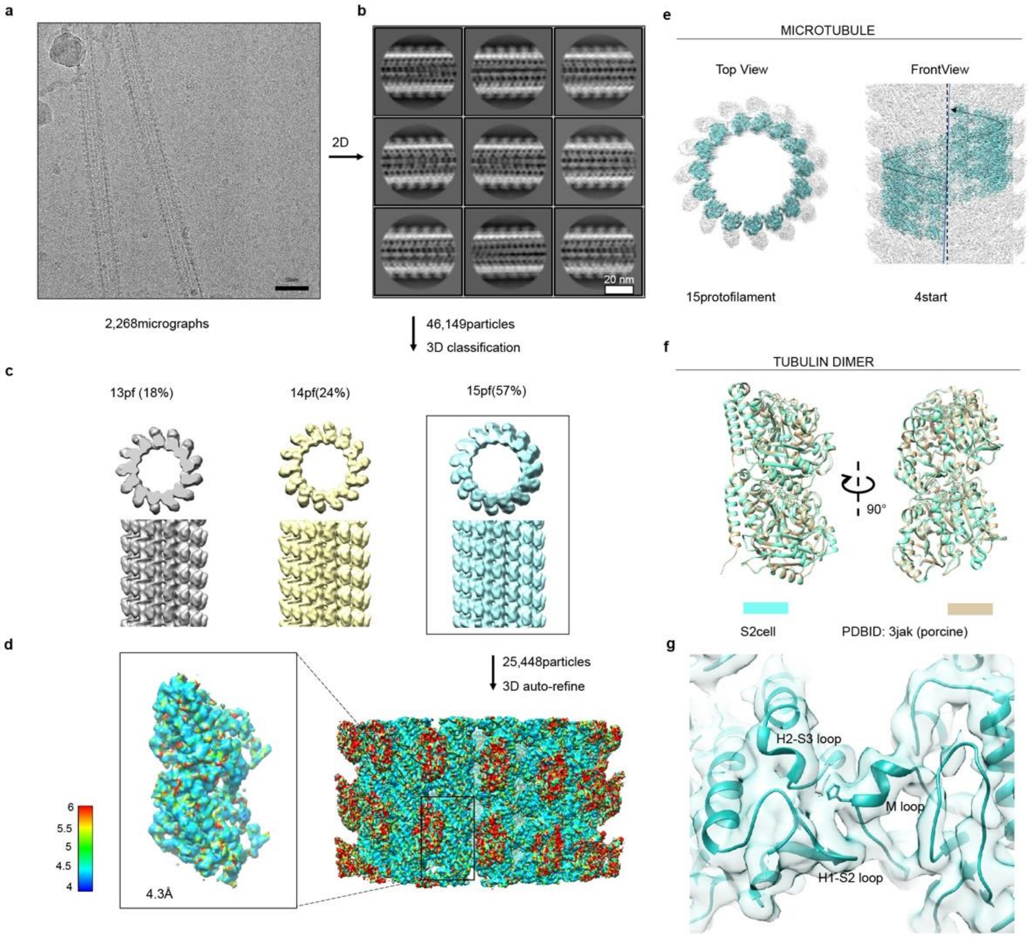

Supplementary Fig. 12 The workflow of Cryo-EM structure reconstruction of S2-GTPγS-MT decorated by KMD.

a A representative Cryo-EM micrograph of GTPγS-MT assembled by *Drosophila* S2 endogenous tubulin decorated by KMD.

b 2D classification results.

c 3D classification shows different MT pfs.

d 3D reconstruction of the 15-pf MT decorated by KMD. The Local resolution map estimation are rendered in the density maps.

e The overall cryo-EM density map of 15-pf MT-KMD complex. Some tubulin atomic models are docked in the map.

f Structural comparison between tubulin heterodimer derived from S2-MT (green) and porcine-MT (tan) (PDBID: 3jak). Structural comparison of tubulin dimer (in the MT lattice) exhibits high conservation between *Drosophila* and Porcine tubulin.

g Cryo-EM density map with the corresponding model around the lateral contacts of "MT-bond". Three major components of α-tubulin (H1-S2 loop, H2-S3 loop and M-loop) are labeled.

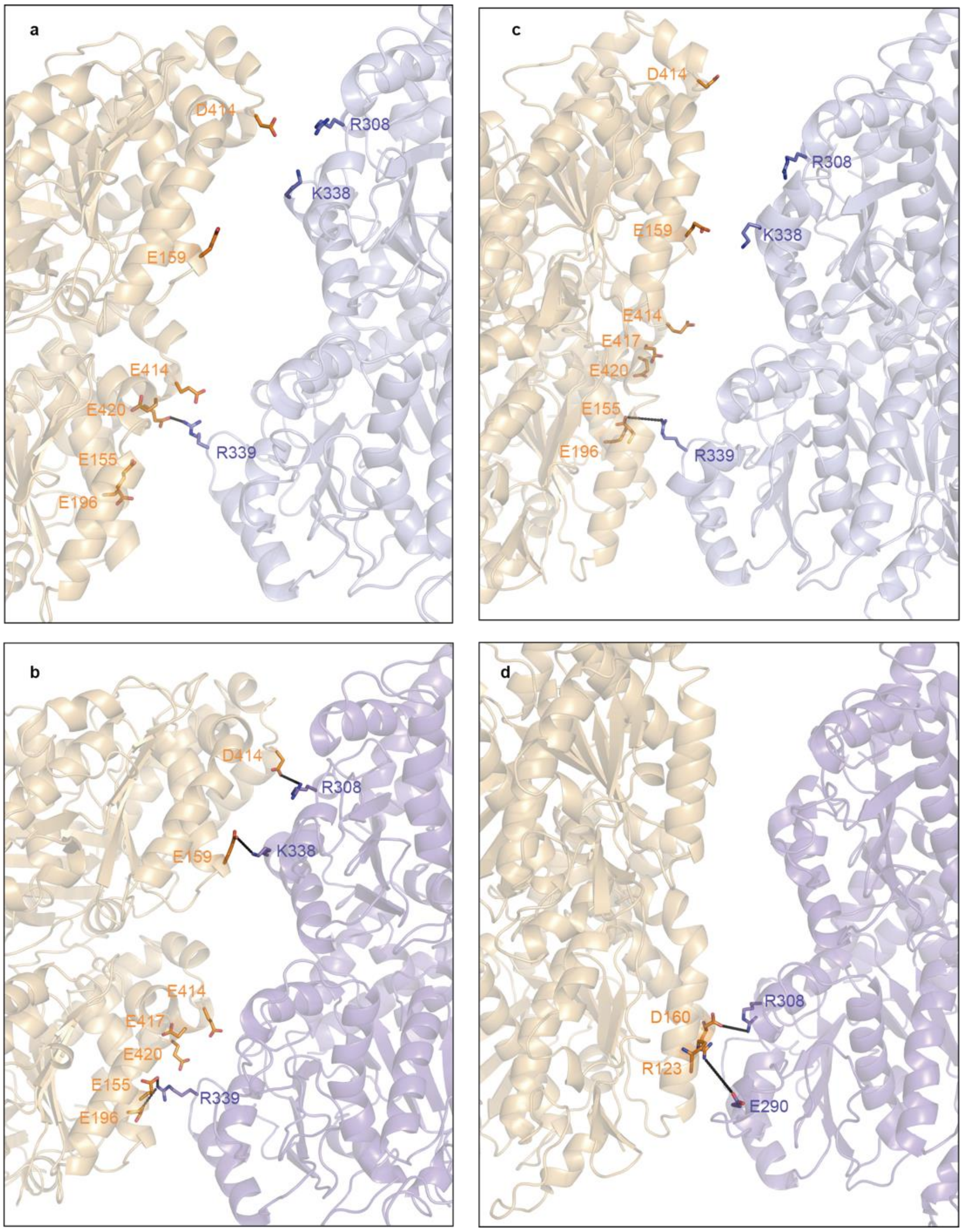

Supplementary Fig. 13 MD simulation snapshots of the lateral interaction during the process of "Tube-to-MT" conversion.

Snapshots of the MD simulations show the lateral interface between two tubulin heterodimers during the process of "Tube-bond Formation" (**a, b**), "Tube-bond Dissociation" (**c**) and "MT-bond Formation" (**d**). The dominant residues are labeled and shown as sticks. Above is β-tubulin, and below is α-tubulin.

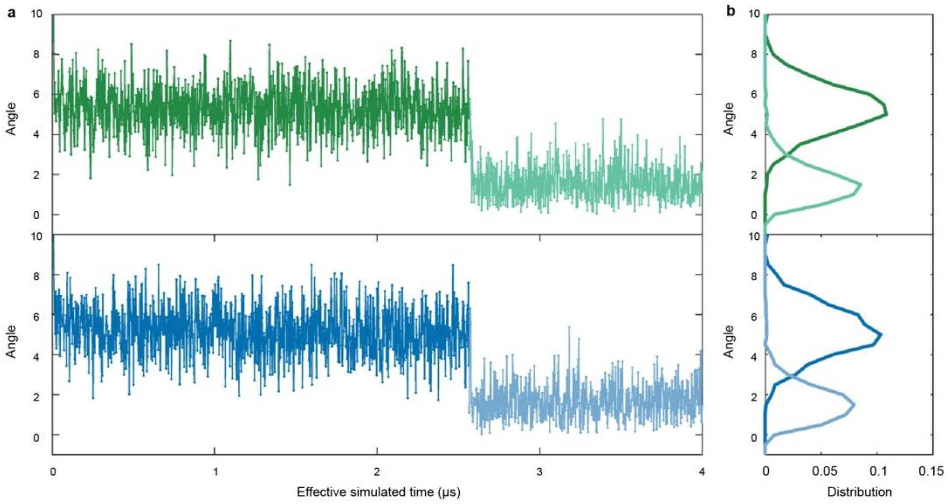

Supplementary Fig. 14 The change of the intra-dimer curvature during the process of "Tube-bond" to "MT-bond".

a The intra-dimer curvature during the process of "MT-bond" formation. The curvature changes when "MT-bond" forms at nearly 2.5 μs. The green curves represent the left tubulin heterodimer in Movie S5 and the blue curves represent the right tubulin heterodimer in Movie S5. The dark and light colors correspond to the intra-dimer curvatures before and after "MT-bond" formation, respectively (same for **b**).

b The intra-dimer curvature distribution.

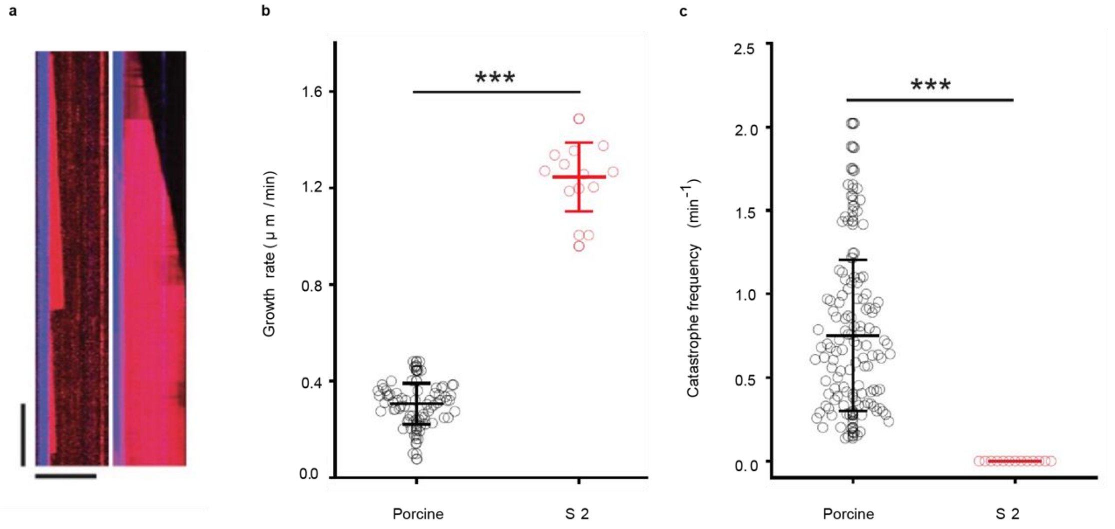

Supplementary Fig. 15 The different dynamics between *Drosophila* S2 and porcine MT

a Representative kymographs showing porcine (left) and *Drosophila* S2 (right) dynamic MTs. Tubulin concentration: 10 μM. Blue: GMPCPP-MT. Red: Dynamic MT. Vertical bar: 100 s. Horizontal bar: 10 μm.

b The growth rate of porcine (black) and Drosophila s2 (red) MT. Tubulin concentration: 10 μM. n = 73 for Porcine MT; n = 13 for Drosophila S2 MT; where n is the number of dynamic MTs. The plot presents mean ± s.d., and individual data points represent one MT. Two-sided unpaired Student's t-test with Bonferroni correction. ***, P < 0.001.

c The catastrophe frequency of porcine (black) and Drosophila s2 (red) MT. Tubulin concentration: 10 μM. n = 122 for Porcine MT; n = 13 for Drosophila S2 MT; where n is the number of dynamic MTs. The plot presents mean ± s.d., and individual data points represent one MT. Two-sided unpaired Student's t-test with Bonferroni correction. ***, P < 0.001.

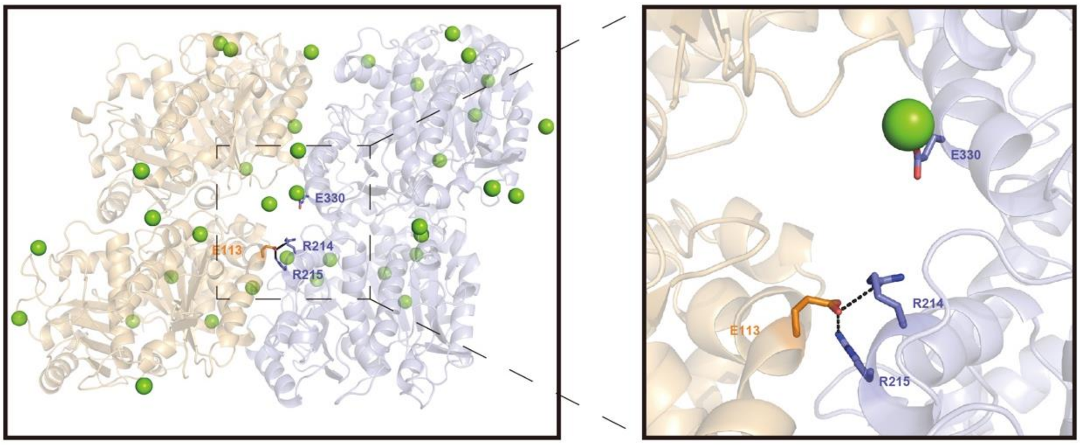

Supplementary Fig. 16 The possible residues coordinated by Mg^{2+} around intra-dimer interface.

Initially, neutralized magnesium ions are placed randomly in simulating structures. After equilibrium, some Mg^{2+} (shown as green spheres) are coordinated to residues located within the intra-dimer interface. In this case, the Mg^{2+} is coordinated to E330, which stabilizes the Tube-bond.

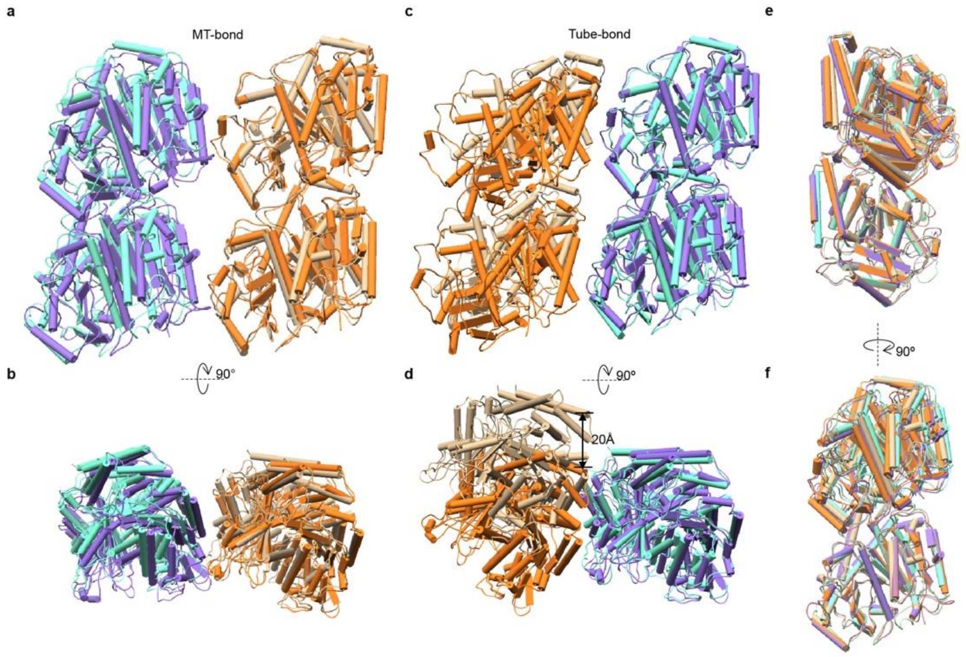

Supplementary Fig. 17 The structural comparison between GTPγS-Tube and GMPCPP-Tube

a-b Structural comparison of "MT-bond" interface between GTPγS-Tube and

GMPCPP-Tube. **a** Side view. **b** Top view. Tubulin structures are superimposed on the right tubulin heterodimer of "MT-bond" interface. The left tubulin heterodimer of "MT-bond" (the right tubulin heterodimer of "Tube-bond" interface) of GTPγS-Tube is colored in purple and the right one is colored in orange. The left tubulin heterodimer of "MT-bond" (the right tubulin heterodimer of "Tube-bond" interface) of GMPCPP-Tube is colored in aquamarine and the right one is colored in tan. (same for **c-f**)

c-d Structural comparison of "MT-bond" interface between GTPγS-Tube and GMPCPP-Tube. **c** Side view. **d** Top view. Tubulin structures are superimposed on the right tubulin heterodimer of "Tube-bond" interface.

e-f Structural comparison of tubulin heterodimers in the GTPγS-Tube and GMPCPP-Tube lattice, superimposed on α-tubulin.

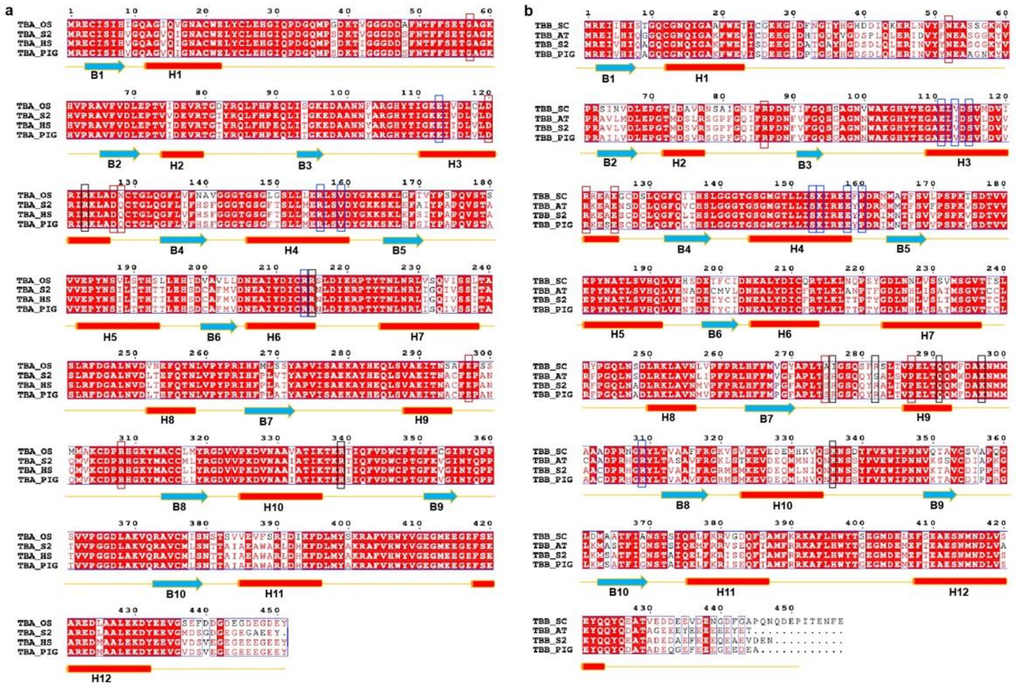

Supplementary Fig. 18 Sequence alignment results among different species.

a α-tubulin sequence alignment result. OS is an abbreviation for *O. sativa* and HS is an abbreviation for *H. sapiens*. A blue box represents residues involved in Tube-bond, a red box represents residues involved in MT-bond, and a black box represents residues involved in both MT-bond and Tube-bond (same for **b**).

b β-tubulin sequence alignment result. SC is an abbreviation for *S. cerevisiae*. AT is an abbreviation for *A. thaliana*.

Supplementary Table 1. The bending angles around the inter-dimer interface

Inter-dimer	Radial bending (º)	Tangential bending (º)	Twist (º)
GDP Class-2 (42%)	-20	11	6
GDP Class-1 (41%)	-22	10	5
GDP Class-4 (12%)	-44	4	-4
GDP state	-23.9±7.7	9.7±2.2	4.3±3.2
GMPCPP Class-4 (53%)	-24	4	6
GMPCPP Class-2 (29%)	-23	7	7
GMPCPP Class-3 (16%)	-41	7	8
GMPCPP state	-26.5±6.4	5.4±1.5	6.6±0.7
6TIS	-17	12	1
6TIY	-16	12	1

The percentage indicates the proportion of particles in each class to the total number of particles.

Supplementary Table 2. Binding free energies of dominant residues of Tube-bond and MT-bond

	Sheet-bond		MT-bond	
	**Total**	**STD**	**Total**	**STD**
**Binding free energy (kcal/mol)**	-26.23	10.33	-45.40	13.47
	**Dominant residues in binding free energy contributions**			
	**Residue**	**Total**	**Residue**	**Total**
**Binding free energy (kcal/mol)**	α:R339	-8.67	α:R339	-9.60
	β:K299	-8.49	β:R88	-8.84
	α:R214	-7.84	β:R123	-7.85
	α:R215	-6.62	α:R308	-5.92
	β:Q293	-6.26	α:Q128	-5.81
	α:E113	-6.07	β:Q293	-5.79
	β:R215	-5.78	α:E297	-5.35
	β:R284	-5.45	α:R123	-5.24
	β:S155	-3.89	α:D120	-4.82
	β:R278	-3.83	β:K299	-4.18
	α:R156	-3.76	β:K338	-3.96
	α:R123	-3.75	β:R278	-3.85
	β:E160	-3.41	β:R284	-3.69
	β:K338	-3.16	α:R215	-2.77
	α:V159	-3.11	β:N54	-2.45
	β:P162	-3.04	α:D127	-2.33
	β:K156	-2.60	β:S277	-2.32
	β:S117	-2.51	β:E127	-2.24
	β:E113	-2.41	α:G57	-2.01
	β:R311	-2.01	β:P289	-1.96

Supplementary Table 3. The bending angles around the intra-dimer interface

Intra-dimer	Radial bending (º)	Tangential bending (º)	Twist (º)
Tubulin in solution	-6.2	7.4	7.4
Tubulin in the Tube	-2.4	-0.9	6.7
Tubulin in the MT	-0.4	0.6	0.2

Supplementary Table 4. Cryo-EM data collection, refinement and validation statistics

	GDP-1	GDP-2	GMPCPP	GTPγS-Tube	GTPγS-MT
**Date collection and processing**					
EM equipment	FEI Titan Krios	FEI Titan Krios	FEI Titan Krios	FEI Titan Krios	FEI Titan Krios
Voltage (kV)	300	300	300	300	300
Detector	Gatan K3	Gatan K3	Gatan K3	Gatan K2	Falcon II
Magnification	81,000	81,000	81,000	22,500	75,000
Electron exposure ($e^-/Å^2$)	50	50	50	50	50
Defocus range (μm)	-1.2 ~ -1.8	-1.2 ~ -1.8	-1.2 ~ -1.8	-0.5 ~ -2.5	-1.0 ~ -2.5
Pixel size (Å)	0.856	0.856	0.856	1.33	1.08
Symmetry imposed	C1	C1	C1	H	H
Particle images (no.)	287,272	143,422	236,436	74,919	25,448
Map resolution (Å)	3.6	3.9	3.5	6.8	4.3
Map sharpening B-factor	-70	-70	-70	-188	-210
FSC threshold	0.143	0.143	0.143	0.143	0.143
**Model composition**					
Non-hydrogen atoms	6812	6812	6846	26920	13460
Protein residues	860	860	864	3392	1696
Ligands	2	2	2	8	4
**R.m.s.deviations**					
Bond lengths (Å)	0.003	0.002	0.002	0.009	0.003
Bond angles (°)	0.663	0.612	0.602	1.86	0.589
**Validation**					
MolProbity Score	1.98	1.83	1.92	2.48	1.93
Clashcore	18.98	18.01	14.95	20.97	13.9
Rotamers outliers(%)	0.00	0.00	0.00	0.00	0.00
**Ramachandran plot**					
Favored (%)	96.72	97.66	96.39	96.53	95.96
Allowed (%)	3.28	2.34	3.61	3.24	4.04
Outliers (%)	0.00	0.00	0.00	0.24	0.00
