# Supplementary material for: Structural insights into the mechanism of GTP initiation of microtubule assembly
Source: Nat Commun. 2023 Sep 25;14:5980. doi: 10.1038/s41467-023-41615-w (PMC10519996; doi:10.1038/s41467-023-41615-w)
Supplement: Supplementary file 3 — Description of Additional Supplementary Files [file 41467_2023_41615_MOESM3_ESM.pdf]

Description of Additional Supplementary Files

Supplementary Movie 1. The 3DVA analysis of GDP-tubulin tetramer in the radial view

This movie demonstrates large structural variation around the inter-dimer interface of GDP-tubulin tetramer in the radial view.

Supplementary Movie 2. The 3DVA analysis of GDP-tubulin tetramer in the tangential view

This movie demonstrates large structural variation around the inter-dimer interface of GDP-tubulin tetramer in the tangential view.

Supplementary Movie 3. The 3DVA analysis of GMPCPP-tubulin tetramer in the radial view

This movie demonstrates large structural variation around the inter-dimer interface of GMPCPP-tubulin tetramer in the radial view.

Supplementary Movie 4. The 3DVA analysis of GMPCPP-tubulin tetramer in the tangential view

This movie demonstrates no structural variation around the inter-dimer interface of GMPCPP-tubulin tetramer in the tangential view.

Supplementary Movie 5. Cryo-EM density map of GTPγS-Tube-KMD docking with tubulin and KMD models

This movie demonstrates the complicated organization of the giant GTPγS-Tube-KMD complex, including Tu-Kin-1/2/3, MT-bond and Tube-bond interfaces.

Supplementary Movie 6. The process of “MT-bond” formation revealed by MD simulations

This movie demonstrates the process of “Tube-bond Formation”, “Tube-bond Dissociation” and “MT-bond Formation”. Top panel: The lateral interactions during the “Tube-bond” (blue) and “MT-bond” (red) interface formation/dissociation process. (Left) Front view. (Right) Top view. Bottom panel: The proportion of native contacts within the “Tube-bond” (blue) and “MT-bond” (red) interface as a function of effective simulated time.
