# Supplementary material for: Structural insights into the mechanism of GTP initiation of microtubule assembly
Source: Nat Commun. 2023 Sep 25;14:5980. doi: 10.1038/s41467-023-41615-w (PMC10519996; doi:10.1038/s41467-023-41615-w)
Supplement: Supplementary file 10 — Reporting Summary [file 41467_2023_41615_MOESM10_ESM.pdf]

nature portfolio

Corresponding author(s): Hong-Wei Wang, Huiying Chu

Last updated by author(s): Aug 30, 2023

Reporting Summary

Nature Portfolio wishes to improve the reproducibility of the work that we publish. This form provides structure for consistency and transparency in reporting. For further information on Nature Portfolio policies, see our Editorial Policies and the Editorial Policy Checklist.

Statistics

For all statistical analyses, confirm that the following items are present in the figure legend, table legend, main text, or Methods section.

n/a	Confirmed	
☒	☐	The exact sample size (*n*) for each experimental group/condition, given as a discrete number and unit of measurement
☒	☐	A statement on whether measurements were taken from distinct samples or whether the same sample was measured repeatedly
☐	☒	The statistical test(s) used AND whether they are one- or two-sided<br>*Only common tests should be described solely by name; describe more complex techniques in the Methods section.*
☒	☐	A description of all covariates tested
☒	☐	A description of any assumptions or corrections, such as tests of normality and adjustment for multiple comparisons
☐	☒	A full description of the statistical parameters including central tendency (e.g. means) or other basic estimates (e.g. regression coefficient) AND variation (e.g. standard deviation) or associated estimates of uncertainty (e.g. confidence intervals)
☐	☒	For null hypothesis testing, the test statistic (e.g. *F*, *t*, *r*) with confidence intervals, effect sizes, degrees of freedom and *P* value noted<br>*Give P values as exact values whenever suitable.*
☒	☐	For Bayesian analysis, information on the choice of priors and Markov chain Monte Carlo settings
☒	☐	For hierarchical and complex designs, identification of the appropriate level for tests and full reporting of outcomes
☒	☐	Estimates of effect sizes (e.g. Cohen's *d*, Pearson's *r*), indicating how they were calculated

Our web collection on statistics for biologists contains articles on many of the points above.

Software and code

Policy information about availability of computer code

Data collection	AutoEMation 2.0, EPU 2.10
Data analysis	cryoSPARC v3.1.0, Relion 3.1, Relion 3.0, MotionCor2_v1.3.1-Cuda101, Phenix 1.19.1-4122, Coot 0.9, pyem 0.5, UCSF Chimera-1.13, AmberTools V6.18.1, Amber18, OpenMM7.4,SMOG2,MDTraj1.9, Python3

For manuscripts utilizing custom algorithms or software that are central to the research but not yet described in published literature, software must be made available to editors and reviewers. We strongly encourage code deposition in a community repository (e.g. GitHub). See the Nature Portfolio guidelines for submitting code & software for further information.

Data

Policy information about availability of data

All manuscripts must include a data availability statement. This statement should provide the following information, where applicable:

- Accession codes, unique identifiers, or web links for publicly available datasets
- A description of any restrictions on data availability
- For clinical datasets or third party data, please ensure that the statement adheres to our policy

Atomic coordinates and their corresponding maps were deposited in RCSB under the accession code: GMPCPP-tubulin in solution (EMD-34077 https://www.ebi.ac.uk/pdbe/entry/emdb/EMD-34077, PDB: 7YSN http://doi.org/10.2210/pdb7YSN/pdb), GDP-1 tubulin heterodimer in solution (EMD-34078 https://www.ebi.ac.uk/pdbe/entry/emdb/EMD-34078, PDB: 7YSO http://doi.org/10.2210/pdb7YSO/pdb), GDP-2 tubulin in solution (EMD-34079 https://www.ebi.ac.uk/

pdbe/entry/emdb/EMD-34079, PDB: 7YSP http://doi.org/10.2210/pdb7YSP/pdb), GTPgammaS-Tube-KMD (EMD-34080 https://www.ebi.ac.uk/pdbe/entry/emdb/EMD-34080, PDB: 7YSQ http://doi.org/10.2210/pdb7YSQ/pdb) and GTPgammaS-MT-KMD (EMD-34081 https://www.ebi.ac.uk/pdbe/entry/emdb/EMD-34081, PDB: 7YSR http://doi.org/10.2210/pdb7YSR/pdb). All other data and materials are available from the corresponding authors upon reasonable request. Previously solved structures used in this study were obtained from the PDB with accession codes: 3JAR https://doi.org/10.2210/pdb3JAR/pdb; 3JAK https://doi.org/10.2210/pdb3JAK/pdb; 6TIS https://doi.org/10.2210/pdb6TIS/pdb; 6TIY https://doi.org/10.2210/pdb6TIY/pdb; 4FFB https://doi.org/10.2210/pdb4FFB/pdb; 4DRX https://doi.org/10.2210/pdb4DRX/pdb. The source data for Fig. 2ijk, Supplementary Fig. 2c, 5a, 14a, 15b and 15c are provided in source file.

Human research participants

Policy information about studies involving human research participants and Sex and Gender in Research.

Reporting on sex and gender	N/A
Population characteristics	N/A
Recruitment	N/A
Ethics oversight	N/A

Note that full information on the approval of the study protocol must also be provided in the manuscript.

Field-specific reporting

Please select the one below that is the best fit for your research. If you are not sure, read the appropriate sections before making your selection.

☒ Life sciences ☐ Behavioural & social sciences ☐ Ecological, evolutionary & environmental sciences

For a reference copy of the document with all sections, see nature.com/documents/nr-reporting-summary-flat.pdf

Life sciences study design

All studies must disclose on these points even when the disclosure is negative.

Sample size	For cryo-EM data, we did not perform sample size calculations. After we believed that the obtained maps could address all conclusions in the manuscript, we stopped processing and collecting data. For GMPCPP-tubulin, we used 236,436 particles to get a reconstruction at 3.5 Å resolution. For GDP-tubulin, 287,272 particles were used to get a reconstruction of GDP-1 at 3.6 Å resolution and 143,422 particles were used to get a reconstruction of GDP-2 at 3.9 Å resolution, respectively. For GTPγS-Tube-KMD, we used 74,919 particles to get a reconstruction at 6.8 Å resolution. For GTPγS-MT, we used 25,448 particles to get the reconstruction at a resolution of 4.3 Å. For Bootstrap sampling, when the size is over 5000, the distributions of GDP and GMPCPP won't change. Consequently, 10000 is chosen as the sample size for the purpose of illustrating the problem adequately.
Data exclusions	The exclusion criteria were not pre-established. 2D and 3D classification yielded multiple classes. Only the particles in the classes that showed clear structural signals were selected, combined and used in the final reconstruction and refinement.
Replication	The negative-staining and TIRF experiments were reliably reproduced at least three times.
Randomization	For structural refinement and model validation, all the particles were randomly chosen. For cryo-EM reconstruction, samples were allocated random, including the particle-motion and structural determination.
Blinding	The study was not blinded since visual inspection of both cryo-EM reconstructions and other experiments was necessary to determine the quality of the data.

Reporting for specific materials, systems and methods

We require information from authors about some types of materials, experimental systems and methods used in many studies. Here, indicate whether each material, system or method listed is relevant to your study. If you are not sure if a list item applies to your research, read the appropriate section before selecting a response.

Materials & experimental systems

n/a	Involved in the study	
☒	☐	Antibodies
☐	☒	Eukaryotic cell lines
☒	☐	Palaeontology and archaeology
☐	☒	Animals and other organisms
☒	☐	Clinical data
☒	☐	Dual use research of concern

Methods

n/a	Involved in the study	
☒	☐	ChIP-seq
☒	☐	Flow cytometry
☒	☐	MRI-based neuroimaging

Eukaryotic cell lines

Policy information about cell lines and Sex and Gender in Research

Cell line source(s)	Drosophila melanogaster S2 cell obtained from Invitrogen.
Authentication	The cell line was obtained from commercial source and doesn't need further authentication.
Mycoplasma contamination	The cell were not tested for mycoplasma.
Commonly misidentified lines (See ICLAC register)	No commonly misidentified cell lines were used in this study.

Animals and other research organisms

Policy information about studies involving animals; ARRIVE guidelines recommended for reporting animal research, and Sex and Gender in Research

Laboratory animals	It was only the separated pig brain that we purchased from Beijing No.5 Meat Processing Factory, and we did not have any animals in the lab.
Wild animals	No
Reporting on sex	No
Field-collected samples	No
Ethics oversight	This study does not require ethical approval.

Note that full information on the approval of the study protocol must also be provided in the manuscript.
